# Supplementary material for: PKCθ Regulates Pituitary Adenoma Bone Invasion by Activating Osteoclast in NF-κB/IL-1β-Dependent Manner
Source: Cancers (Basel). 2023 Mar 6;15(5):1624. doi: 10.3390/cancers15051624 (PMC10001016; doi:10.3390/cancers15051624)
Supplement: Supplementary file 1 [file cancers-15-01624-s001.zip › cancers-2211653-supplementary.pdf]

Supplementary Materials: PKCθ regulates pituitary adenoma bone invasion by activating osteoclast in NF-κB/IL-1β dependent manner

Quanji Wang, Zhuowei Lei, Zihan Wang, Qian Jiang, Zhuo Zhang, Xiaojin Liu, Biao Xin, Sihan Li, Xiang Guo, Yanchao Liu, Xingbo Li, Kai Shu, Huaqiu Zhang, Yimin Huang and Ting Lei

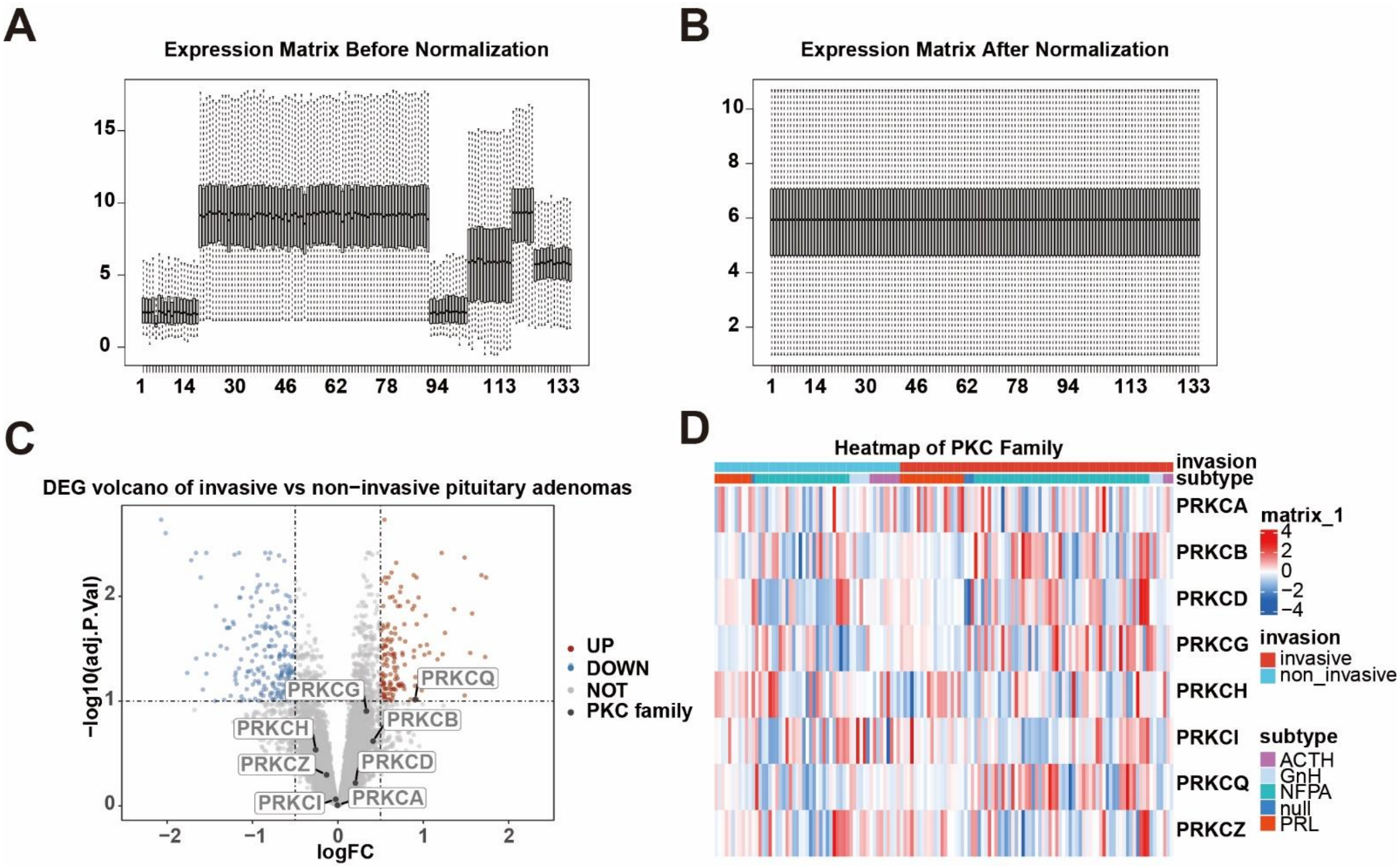

Figure S1. Bioinformatics data collation and analysis. (**A**, **B**) Boxplots show the intensity of the gene expression data before (**A**) and after (**B**) batch effect removal. (**C**) Volcano plot of DEGs between BIPAs versus non-BIPAs, emphasizing genes of PKC family.(**D**) Heatmap of gene expression data of PKC family in PAs.

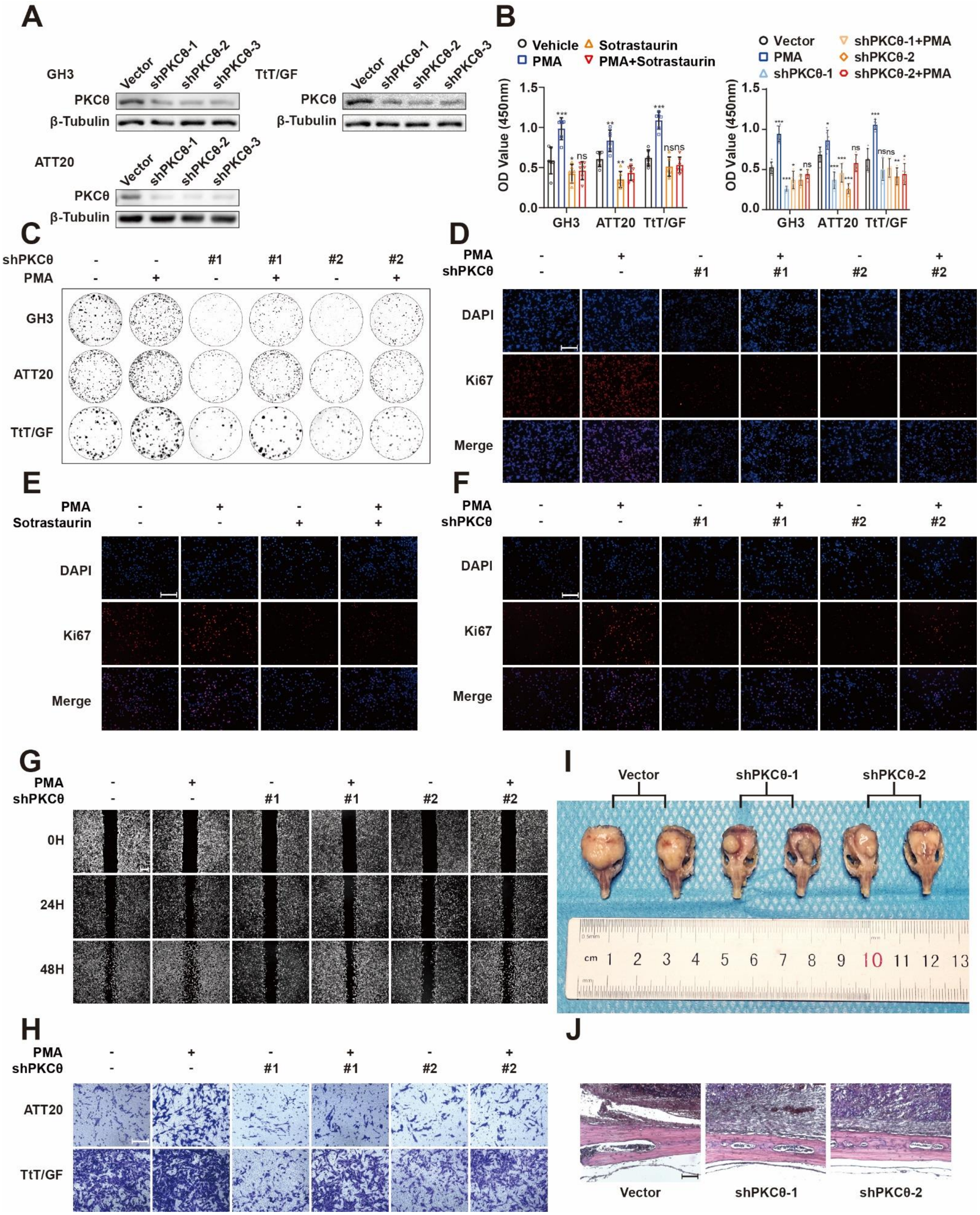

Figure S2. PKCθ in proliferation, migration and bone invasion of PAs. (**A**) WB verification of shPKCθ efficiency in PAs cell lines. (**B**) The CCK-8 cell viability assay of PAs cell lines treated by PMA and Sotrastaurin, left. The CCK-8 cell viability assay of shPKCθ in PAs cell lines, right. (*** P<

0.001; ** P< 0.01; * P< 0.05; NS P≥0.05) (**C**) The colony formation of shPKCθ in PAs cell lines. (**D**) The KI-67 staining of shPKCθ in GH3 cells (× 200; scale bar, 200 μm). (**E**) The KI-67 staining of TtT/GF treated by PMA and Sotrastaurin (× 200; scale bar, 200 μm). (**F**) The KI-67 staining of shPKCθ in TtT/GF cells (× 200; scale bar, 200 μm). (**G**) The wound healing assay of shPKCθ in TtT/GF cells (× 100; scale bar, 200 μm). (**H**) The transwell assay of shPKCθ in ATT20 and TtT/GF cells (× 200; scale bar, 200 μm). (**I**) Photographs of ATT20 calvaria xenograft tumor specimens in groups of vector and shPKCθ. (**J**) HE staining exhibited bone destruction of ATT20 tumors in groups of vector and shPKCθ (× 40; scale bar, 500 μm).

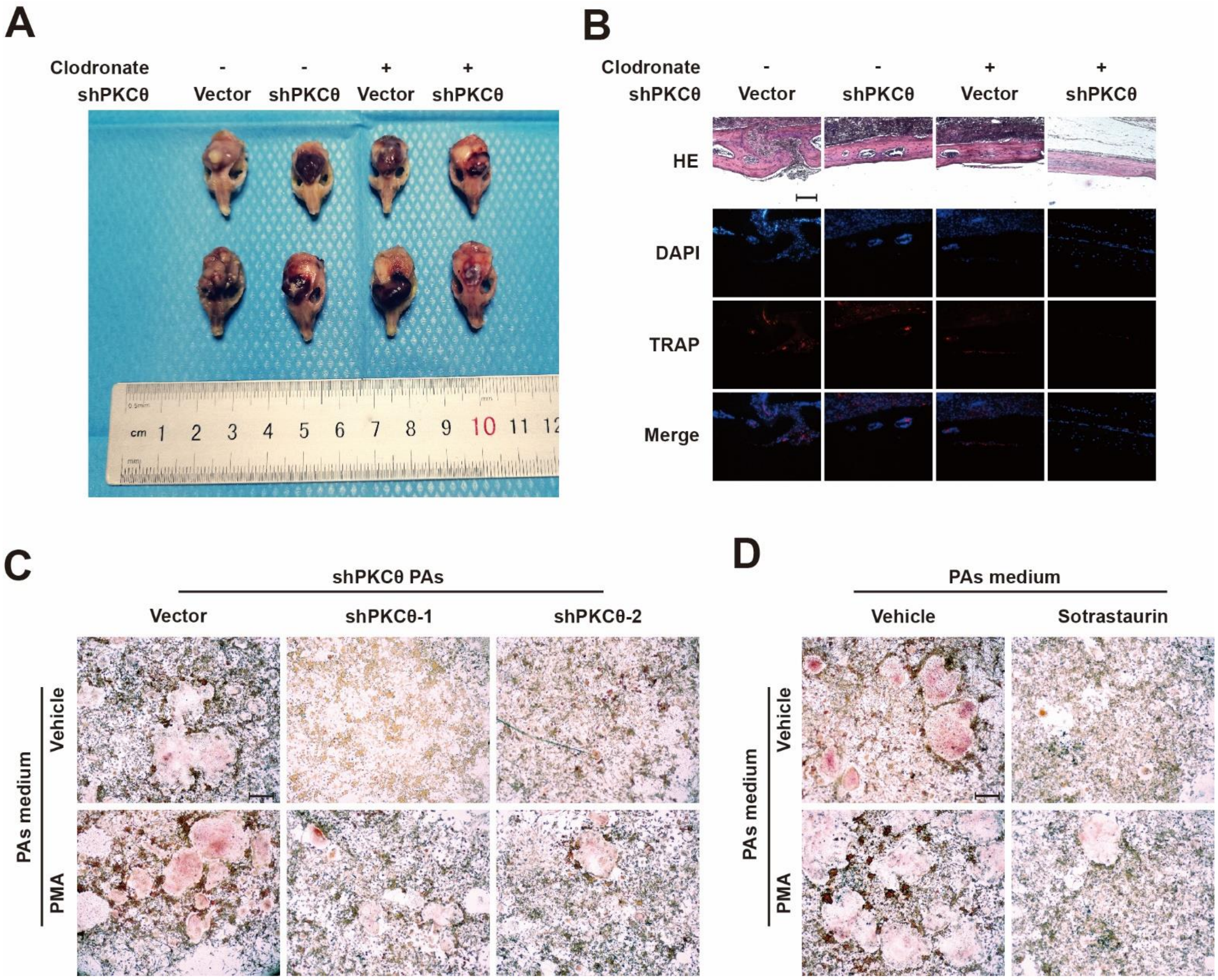

Figure S3. PAs induce macrophage-osteoclast differentiation via the paracrine pathway. (**A**) Photographs of ATT20 calvaria xenograft tumor specimens in treatment of Clodronate Liposomes and shPKCθ. (**B**) HE staining and immunofluorescence of TRAP+ cells in the tumor-bone junction area (× 200; scale bar, 200 μm). (**C**) TRAP staining of RAW264.7 administrated of 30ng/ml RANKL and CM of ATT20 cells in groups of vector and shPKCθ(× 40; scale bar, 500 μm). (**D**) TRAP staining of RAW264.7 administrated of 30ng/ml RANKL and CM of ATT20 cells in treatment of Sotrastaurin and PMA (× 40; scale bar, 500 μm).

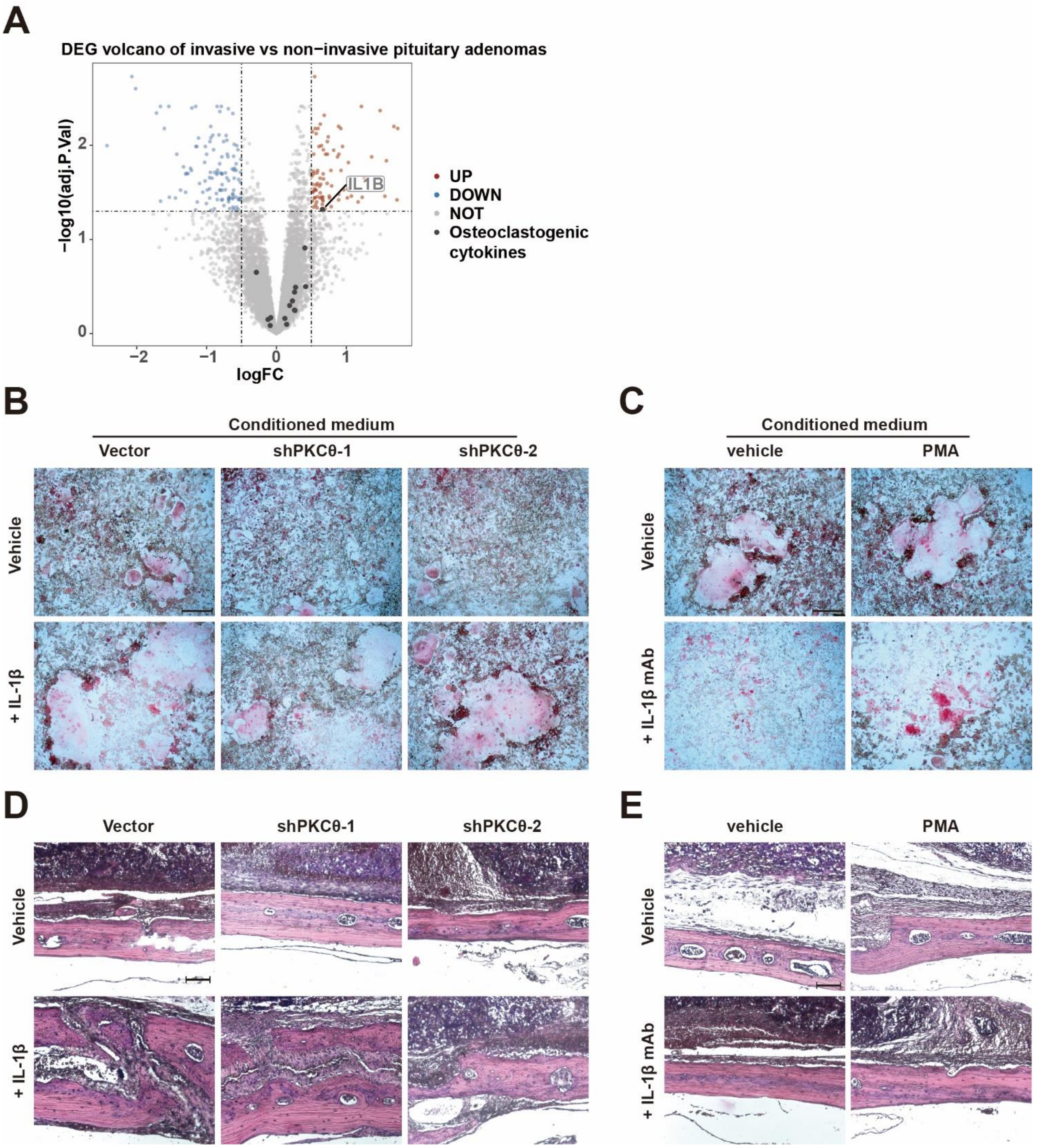

Figure S4. Pituitary tumors promote bone invasion by releasing IL1b. (**A**) Volcano plot showing the distribution of DEGs of osteogenic cytokines in BIPAs non-BIPAs (The data analyzed are described in Fig. 2A). (**B**) TRAP staining of RAW264.7 administrated of IL-1β recombinant protein and CM of ATT20 cells in groups of vector and shPKCθ (× 40; scale bar, 500 μm). (**C**) TRAP staining of RAW264.7 administrated of IL-1β mAb and CM medium of ATT20 cells in treatment of PMA (× 40; scale bar, 500 μm). (**D**) HE staining exhibited bone destruction of xenograft tumor of ATT20 while shPKC and administration of IL-1β recombinant protein(× 200; scale bar, 100 μm). (**E**) HE staining exhibited bone destruction of xenograft tumor of ATT20 while administrated of IL-1β mAb and PMA (× 200; scale bar, 100 μm).

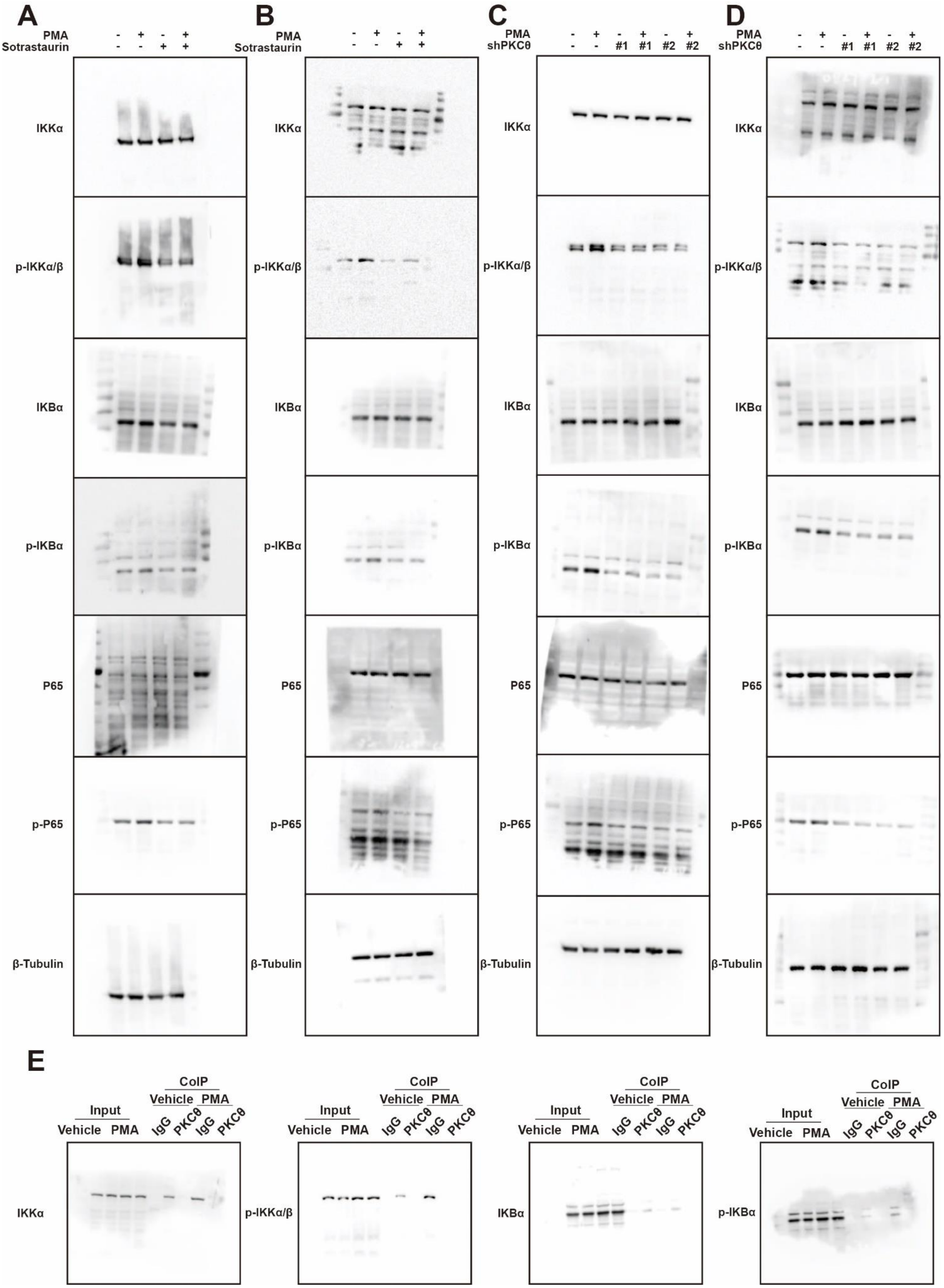
A
PMA
Sotrastaurin
IKKα
p-IKKα/β
IKBα
p-IKBα
P65
p-P65
β-Tubulin
B
PMA
Sotrastaurin
IKKα
p-IKKα/β
IKBα
p-IKBα
P65
p-P65
β-Tubulin
C
PMA
shPKCθ
#1
#2
IKKα
p-IKKα/β
IKBα
p-IKBα
P65
p-P65
β-Tubulin
D
PMA
shPKCθ
#1
#2
IKKα
p-IKKα/β
IKBα
p-IKBα
P65
p-P65
β-Tubulin
E
Input
CoIP
Vehicle PMA
IgG PKCθ IgG PKCθ
IKKα
p-IKKα/β
IKBα
p-IKBα

Figure S5. Complete picture of WB. (**A**) Complete picture of **Figure 5K.** (**B**) Complete picture of **Figure 5L.** (**C**) Complete picture of **Figure 5M.** (**D**) Complete picture of **Figure 5N.** (**E**) Complete picture of **Figure 5P.**

Table S1. Primers used in qPCR.

Gene Name	Forward Primer	Reverse Primer
Rat PRKCQ	5′-CTCGTGGAACGCAACTAGGC-3′	5′-AGAGCAGAACGTGGGTTGAG-3′
Mouse PRKCQ	5′-CTCTCACAGCAGCCCTTGAA-3′	5′-GCGACATCCTTACCCTTCCC-3′
Rat GAPDH	5′-GCATCTTCTTGTGCAGTGCC-3′	5′-GATGGTGATGGGTTTCCCGT-3′
Mouse GAPDH	5′-GCCTCCTCCAATTCAACCCT-3′	5′-CTCGTGGTTCACACCCATCA-3′
Human IL1B	5′-ATGATGGCTTATTACAGTGGCAA-3′	5′-GTCGGAGATTCGTAGCTGGA-3′
Human IL6	5′-ACTCACCTCTTCAGAACGAATTG-3′	5′-CCATCTTTGGAAGGTTCAGGTTG-3′
Human TNF	5′-CCTCTCTCTAATCAGCCCTCTG-3′	5′-GAGGACCTGGGAGTAGATGAG-3′
Human IL13	5′-CCTCATGGCGCTTTTGTTGAC-3′	5′-TCTGGTTCTGGGTGATGTTGA-3′
Human IL5	5′-TGGAGCTGCCTACGTGTATG-3′	5′-TTCGATGAGTAGAAAGCAGTGC-3′
Human IFNB1	5′-ATGACCAACAAGTGTCTCCTCC-3′	5′-GGAATCCAAGCAAGTTGTAGCTC-3′
Human LIF	5′-CCAACGTGACGGACTTCCC-3′	5′-TACACGACTATGCGGTACAGC-3′
Human CSF1	5′-TGGCGAGCAGGAGTATCAC-3′	5′-AGGTCTCCATCTGACTGTCAAT-3′
Human CXCL8	5′-ACTCCAAACCTTTCCACCCC-3′	5′-TTCTCAGCCCTCTTCAAAAACTT-3′
Human IL4	5′-CCAACTGCTTCCCCCTCTG-3′	5′-TCTGTTACGGTCAACTCGGTG-3′
Human IFNG	5′-TCGGTAACTGACTTGAATGTCCA-3′	5′-TCGCTTCCCTGTTTTAGCTGC-3′
Human IL18	5′-TCTTCATTGACCAAGGAAATCGG-3′	5′-TCCGGGGTGCATTATCTCTAC-3′
Human IL10	5′-GACTTTAAGGGTTACCTGGGTTG-3′	5′-TCACATGCGCCTTGATGTCTG-3′
Human TNFSF11	5′-CAACATATCGTTGGATCACAGCA-3′	5′-GACAGACTCACTTTATGGGAACC-3′
Human IL15	5′-TTGGGAACCATAGATTTGTGCAG-3′	5′-GGGTGAACATCACTTTCCGTAT-3′
Human IL7	5′-TTGGACTTCCTCCCCTGATCC-3′	5′-TCGATGCTGACCATTAGAACAC-3′
Human IL1A	5′-TGGTAGTAGCAACCAACGGGA-3′	5′-ACTTTGATTGAGGGCGTCATTC-3′
Human CCL2	5′-CAGCCAGATGCAATCAATGCC-3′	5′-TGGAATCCTGAACCCACTTCT-3′
Human IL17A	5′-TCCCACGAAATCCAGGATGC-3′	5′-GGATGTTCAGGTTGACCATCAC-3′
Human IL23A	5′-CTCAGGGACAACAGTCAGTTC-3′	5′-ACAGGGCTATCAGGGAGCA-3′
Human IL11	5′-CGAGCGGACCTACTGTCCTA-3′	5′-GCCCAGTCAAGTGTCAGGTG-3′
Human IL2	5′-TACAAGAACCCGAAACTGACTCG-3′	5′-ACATGAAGGTAGTCTCACTGCC-3′
Human IFNA1	5′-GCCTCGCCCTTTGCTTTACT-3′	5′-CTGTGGGTCTCAGGGAGATCA-3′
Human CCL3	5′-AGTTCTCTGCATCACTTGCTG-3′	5′-CGGCTTCGCTTGGTTAGGAA-3′
Human CSF2	5′-TCCTGAACCTGAGTAGAGACAC-3′	5′-TGCTGCTTGTAGTGGCTGG-3′
Human CCL5	5′-CCAGCAGTCGTCTTTGTCAC-3′	5′-CTCTGGGTTGGCACACACTT-3′
Human IL34	5′-CCTGGCTGCGCTATCTTGG-3′	5′-AGTGTTTCATGTACTGAAGTCGG-3′
Human TGFB1	5′-GGCCAGATCCTGTCCAAGC-3′	5′-GTGGGTTTCCACCATTAGCAC-3′
